# Excessive addition split peak formed by the non-templated nucleotide addition property of *Taq* DNA polymerase after PCR amplification

**DOI:** 10.3389/fbioe.2023.1180542

**Published:** 2023-04-27

**Authors:** Yongsong Zhou, Fan Bo, Tian Tian, Buling Wu, Bofeng Zhu

**Affiliations:** ^1^ Shenzhen Stomatology Hospital (Pingshan), Southern Medical University, Shenzhen, Guangdong, China; ^2^ Guangzhou Key Laboratory of Forensic Multi-Omics for Precision Identification, School of Forensic Medicine, Southern Medical University, Guangzhou, Guangdong, China; ^3^ Key Laboratory of Shaanxi Province for Craniofacial Precision Medicine Research, College of Stomatology, Xi’an Jiaotong University, Xi’an, Shanxi, China

**Keywords:** non-template addition, *Taq* DNA polymerase, artifact, split peak, capillary electrophoresis

## Abstract

Because of its non-template addition feature, *Taq* DNA polymerase can catalyze one or more extra nucleotides onto the 3′ terminus of PCR products. An extra peak is observed at DYS391 locus after the PCR products stored for 4 days at 4°C. To explore the formation mechanism of this artifact, PCR primers and amplicon sequences of Y-STR loci are analyzed, furthermore, PCR products storage conditions and termination of PCR are discussed. The extra peak is a + 2 addition product, which we call excessive addition split peak (EASP). The most significant difference between EASP and the incomplete addition of adenine product is that the size of EASP is about one base larger than the true allele, and the EASP locates on the right side of the real allelic peak. The EASP cannot be eliminated by increasing loading mixture volume and conducting heat denaturation prior to electrophoresis injection. However, the EASP is not observed when the PCR is terminated with ethylenediaminetetraacetic acid or formamide. These findings suggest that formation of EASP is a result of 3′ end non-template extension by *Taq* DNA polymerase, rather than being the result of DNA fragment secondary structure produced under a suboptimal electrophoresis condition. In addition, the EASP formation is affected by the primer sequences and the storage conditions of PCR products.

## 1 Introduction

DNA polymerases, a group of enzymes that widely exist in cells of almost all living organisms, which not only play a fundamental role *in vivo* during the processes of DNA replication, recombination and repair, but are also universally used *in vitro* for DNA manipulation, such as polymerase chain reaction (PCR), cloning, DNA sequencing and site-directed mutagenesis (Aschenbrenner and Marx, 2017). Since the purification and characterization of a thermostable DNA polymerase from *Thermus aquaticus* (*Taq*) (Chien et al., 1976), the applications of *Taq* DNA polymerase have been dramatically expanded in molecular biology, especially in PCR-related methods (Terpe, 2013), because of its core competence to still maintain relatively high catalytic activity and stability after multiple rounds of thermal cycling at high temperatures (Saiki et al., 1985; Mullis and Faloona, 1987). In PCR amplification reaction, although *Taq* DNA polymerase normally requires coding information provided by template strands to add deoxyribonucleotide triphosphate (dNTP) one by one onto the 3′ hydroxyl ends of primers, it has the capacity to perform nucleotide addition without absolutely relying on the instruction of DNA templates (Clark, 1988).

It is a well-known fact that several types of DNA polymerases from procaryotic to eucaryotic organisms can plus one or more base overhangs to the 3′ terminals of double-stranded PCR products (Clark et al., 1987; Clark, 1988; Garcia et al., 2004). On the bright side, the property of non-templated nucleotide addition offers many advantages for the application of PCR methods in biology, for example, the famous TA cloning strategy (Green and Sambrook, 2021). However, on the other side, some drawbacks can be caused by the intrinsic non-template-directed DNA synthesis characteristic of *Taq* DNA polymerase when it is used for microsatellite genotyping analysis. The shoulder peak or split peak in a multiplex PCR product is formed by incomplete non-template nucleotide addition, which might make the identification of alleles inaccurate and the interpretation of typing results unclear or incorrect (Smith et al., 1995; Butler, 2015).

In the re-detection of our recently obtained Y-chromosomal short tandem repeat (Y-STR) PCR amplified products, we found that a class of unexpected experimental artifacts were observed at loci DYS391, DYS576 and DYS458 in a recently released commercial Y-STR kit. From the size and location of the extra peak in the genotyping profile, these artificial peaks were not exactly the same as the split peak defined by Butler (Butler, 2015). According to the electrophoresis results which performed on Applied Biosystems 3500xL Genetic Analyzer, the observed artifact in size was approximately one base longer than the identified main peak. So, it located on the right side of the main allelic peak.

In this study, the characteristics of these artifacts and the environmental conditions for their formation will be discussed. The primer sequences of loci and the PCR products stored at different temperatures and durations are analyzed. Furthermore, the reverse primers of those Y-STR loci preferred to generate artifact are modified purposefully to explore the probable formation mechanisms of these accident PCR segments. The artificial peaks are + 2 addition products that formed by non-templated nucleotide addition of *Taq* DNA polymerase under non-PCR amplification conditions.

## 2 Materials and methods

### 2.1 PCR amplification conditions

PCR amplification reactions of the AGCU Y43CS kit (Y43CS; AGCU Biotech Co., Ltd., Wuxi, China) were prepared in a 10.0 μL total volume, consisting of 4.0 μL of master mix, 3.0 μL of sdH_2_O, 2.0 μL of primer sets and 1.0 μL of Control DNA 9948 (1.0 ng/μL; Promega Corporation, Madison, WI, United States). Thermal cycling parameters were set up as the manufacturer’s recommendation: enzyme activation at 95°C for 5 min; 30 cycles of 94°C for 10 s, 60°C for 1 min, and 68°C for 1 min; followed by a final extension step at 60°C for 10 min. Thermal cycling conditions and PCR amplification of the VeriFiler^™^ Plus kit (VFP; Thermo Fisher Scientific, Waltham, MA United States) were performed as the method reported by Green et al. (Green et al., 2021). Thermal cycling of both kits were carried out on the GeneAmp^™^ PCR system 9700 (Thermo Fisher Scientific) with a gold-plated silver block and ‘Max’ mode.

### 2.2 Variations of *Taq* DNA polymerase and final extension time

The Y43CS master mix includes several key components: a hot start *Taq* DNA polymerase, dNTPs, salts, buffer, betaine, and bovine serum albumin. To test the effect of *Taq* DNA polymerase on the formation of artifacts in the Y43CS, 1 U, 2 U, 3 U, 4 U, 5 U and 6 U of *Taq* DNA polymerase (AGCU Biotech) were added into a 10.0 μL of PCR reaction volume, respectively. The amplification was performed on the 9700 PCR thermal cycler according to the standard PCR cycling conditions. Several different final extension time were examined including 0 min, 10 min, 30 min, 60 min, 90 min and 120 min to evaluate their effects on Y43CS amplification products.

### 2.3 PCR product electrophoresis and genotyping

The PCR products of both kits were analyzed on the Applied Biosystems 3500xL Genetic Analyzer, and the raw data were sized and genotyped by Data Collection software 3.1.2. AGCU A6Dye matrix standard (AGCU Biotech), and DS-37 matrix standard (Thermo Fisher Scientific) were used for spectral calibration in 3500xL instrument to Y43CS and VFP, respectively. As far as the Y43CS was concerned, loading samples were prepared for capillary electrophoresis (CE) via adding 1.0 μL of PCR amplification product or Y43CS allelic ladder to 12.0 μL of loading mixture, which was composed of 1.0 μL of AGCU Marker SIZ 600 Size Standard (AGCU Biotech) and 11.0 μL of deionized Hi-Di^™^ formamide (Thermo Fisher Scientific). Loading samples were separated under the default injection and electrophoresis conditions of the 3500xL Genetic Analyzer. Samples preparation and electrophoresis parameters setting of the VFP were consistent with those of described in previous literature (Green et al., 2021). All of the loading samples were heated at 95°C for 3 min and immediately chilled on ice-water mixture for 2 min, prior to electrophoresis. GeneMapper^™^ *ID-X* software v1.6 (Thermo Fisher Scientific) was used for electropherogram analysis with an analytical threshold of 150 relative fluorescence unit (RFU).

### 2.4 PCR product storage and termination of the PCR

In order to investigate the effects of storage temperature and time for the extra peak formation of PCR products, a series of PCR products storage experiments were conducted under different conditions. The detailed information was described in Supplementary Table S1. In brief, three different storage temperatures 4°C, − 20°C and room temperature were tested, and the storage time was varied from 1 to 10 days.

Two methods were used to terminate the PCR amplification reaction, when the PCR was finished in a thermal cycler. In the first method, various volumes of ethylenediaminetetraacetic acid (EDTA; Sigma-Aldrich, Shanghai, China) solutions with a concentration of 10.0 mmol/L were added into the PCR amplification products of the Y43CS to a final concentration of 1.0 mmol/L, 2.0 mmol/L, 3.0 mmol/L and 4.0 mmol/L, respectively. In the second method, 30.0 μL of deionized Hi-Di^™^ formamide was added to the 10.0 μL of PCR reaction volume of the Y43CS immediately after PCR amplification reaction. And then, the PCR products and EDTA/formamide mixtures were stored at different conditions (Supplementary Table S2), and detected on 3500xL Genetic Analyzer for the observation of artificial peak.

### 2.5 Primer and reverse primer modification

Individual forward and reverse primers for each single locus and the original reverse primer sequences of loci DYS391, DYS576, DYS458, DYS19, and DYS481 in the Y43CS were generously provided by AGCU Biotech. The nucleotides at the 5′ end of the reverse primers of these five loci were artificially modified, as shown in Table 1 for details. Subsequently, the modified primer sequences were analyzed using Primer Premier 5.0 software, and the primer oligonucleotides were synthesized by Sangon Biotech Co., Ltd. (Shanghai, China). Each chemically resynthesized reverse primer of every abovementioned locus was mixed with its original forward primer in the Y43CS at 1.0 μmol/L concentration. The quality of each mixed primer pairs was firstly confirmed by singleplex PCR reactions with a 10.0 μL total volume, and then tested by multiplex PCR amplification reactions with standard Y43CS PCR conditions.

**TABLE 1:** General information of the original and revised reverse primers of loci DYS391, DYS576, DYS458, DYS19 and DYS481.

Locus	Reverse primer sequences (5′ to 3′)^a^	Primer length (nt)	GC content (%)	T_m_ (°C)	Description of primers or changes in primers
DYS391	**AT**GCCATAGA…	22	50.0	62.3	The original reverse unlabeled primer of locus DYS391 used in the Y43CS
	**TT**GCCATAGA…	22	50.0	63.3	The initial nucleotide “A” at the 5′ end of original reverse primer was replaced by “T”
	**CT**GCCATAGA…	22	54.5	63.0	The initial nucleotide “A” at the 5′ end of original reverse primer was replaced by “C"
	**GT**GCCATAGA…	22	54.5	62.9	The initial nucleotide “A” at the 5′ end of original reverse primer was replaced by “G"
	GCCATAGAGG…	20	55.0	59.5	The first two nucleotides “AT” were purposely removed from the 5′ end of the original reverse primer of locus DYS391 in Y43CS
DYS576	**AT**TACATAGC…	28	35.7	64.3	The original reverse unlabeled primer of locus DYS576 used in the Y43CS
	**TT**TACATAGC…	28	35.7	65.1	The initial nucleotide “A” at the 5′ end of original reverse primer was replaced by “T”
	**CT**TACATAGC…	28	39.3	64.9	The initial nucleotide “A” at the 5′ end of original reverse primer was replaced by “C”
	**GT**TACATAGC…	28	39.3	64.8	The initial nucleotide “A" at the 5′ end of original reverse primer was replaced by “G”
	TACATAGCAA…	26	38.5	63.3	The first two nucleotides “AT” were purposely removed from the 5′ end of the original reverse primer of locus DYS576 in Y43CS
DYS458	**AT**CCCAAAGT…	23	39.1	65.6	The original reverse unlabeled primer of locus DYS458 used in the Y43CS
	**TT**CCCAAAGT…	23	39.1	66.5	The initial nucleotide “A” at the 5′ end of original reverse primer was replaced by “T”
	**CT**CCCAAAGT…	23	43.5	66.2	The initial nucleotide “A" at the 5′ end of original reverse primer was replaced by “C"
	**GT**CCCAAAGT…	23	43.5	66.2	The initial nucleotide “A" at the 5′ end of original reverse primer was replaced by “G"
	CCCAAAGTTC…	21	42.9	64.0	The first two nucleotides “AT” were purposely removed from the 5′ end of the original reverse primer of locus DYS458 in Y43CS
DYS19	GGGCACCAGG…	20	50.0	60.0	The original reverse unlabeled primer of locus DYS19 used in the Y43CS
	**AT**GGGCACCA…	22	45.5	62.8	Two nucleotides “AT” were artificially added to the 5′ end of the original reverse primer of locus DYS19 in Y43CS
DYS481	CCCACAACCC…	18	55.6	61.3	The original reverse unlabeled primer of locus DYS481 used in the Y43CS
	**AT**CCCACAAC…	20	50.0	63.3	Two nucleotides “AT” were artificially added to the 5′ end of the original reverse primer of locus DYS481 in Y43CS

Notes.

^a^ Primer sequences were not fully displayed for the purpose of protecting commercial confidentiality.

## 3 Results

### 3.1 Characteristics of the EASP under various storage conditions

**FIGURE 1:**
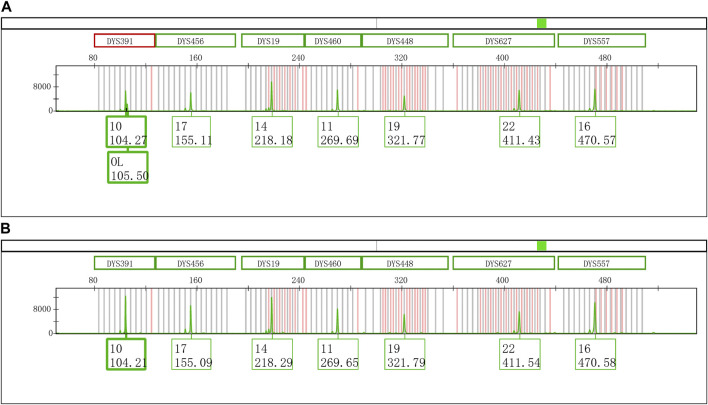
Electrophoretogram results of the green dye channel in Y43CS. **(A)** The allelic peak of locus DYS391 with an extra peak of 105.50 bp. The extra peak was located on the right side of allele 10 at locus DYS391. The peak heights of allele 10 and extra peak were 6756 RFU and 2296 RFU, respectively, and the peak height ratio of extra peak to allele 10 was 0.3398. **(B)** The typical allelic peak of locus DYS391. The size of peak was 104.21 bp, and labeled as allele 10 with a height of 12496 RFU.

An extra peak was accidentally observed at the DYS391 locus during the second CE of Y43CS amplification products on the Applied Biosystems 3500xL Genetic Analyzer (Figure 1A). However, inexplicably, this artifact was not found when the same PCR amplified segments were firstly electrophoresed on the same instrument under the identical electrophoresis conditions (Figure 1B). The only difference between the two electrophoresis tests was that the PCR products used in the second electrophoresis were stored in a refrigerator at 4°C for 4 days. Interestingly, we found that the extra peaks observed in our experimental results were not identical with the split peak defined by Butler (Butler, 2015), although these two kinds of artifacts were similar in some extent. The abnormal short tandem repeat (STR) peak is composed of two parts, an extra peak with a size of *n* and a main peak with a size of *n* + 1, which is described by Butler as incomplete 3′ A nucleotide addition. Unlike the characteristics of split peak defined in the previous report, the size of extra peak observed in this study is about one base larger than the true allele and located on the right side of the true allelic peak, which we called it excessive addition split peak (EASP) here.

**FIGURE 2:**
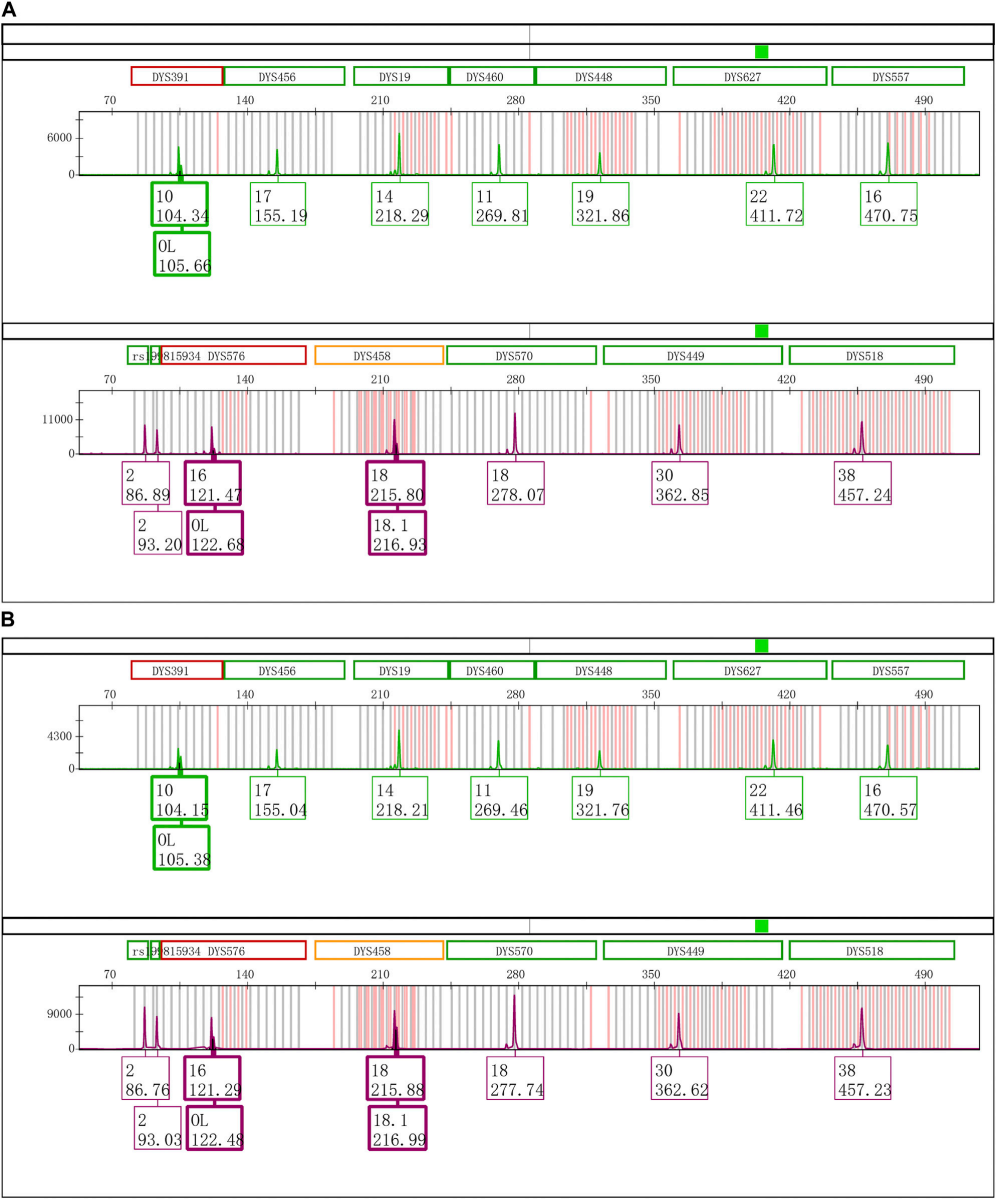
Electrophoretogram results of the green and purple dye channels in Y43CS. **(A)** The extra peaks appeared at loci DYS391, DYS576 and DYS458 after the PCR products stored 6 days at 4°C. The peak height ratios of extra peaks to true alleles at the three loci were 0.3413, 0.2037 and 0.3089, respectively. **(B)** The extra peaks appeared at loci DYS391, DYS576 and DYS458 after the PCR products stored 1 day at room temperature. The peak height ratios of extra peaks to true alleles at the three loci were 0.6061, 0.3907 and 0.6012, respectively.

The Control DNA 9948 was reamplified with Y43CS and VFP kits, respectively. After that the amplification products were stored at 4°C, room temperature, and − 20°C to observe whether the EASP would reappear. The results showed that after the PCR products were stored at 4°C for 6 days, the EASPs were also observed at DYS576 and DYS458 loci, not just at DYS391 locus in the Y43CS (Figure 2A). By contrast, EASPs were observed at these loci after the PCR products were placed at room temperature for only 1 day in a dark place (Figure 2B). Generally, with the increase of storage time, heights of the artificial peaks and the main peaks gradually increased and decreased, respectively. In other words, more and more PCR products were converted to excessive non-templated nucleotide addition products, as storage time increased. Moreover, the increasing number of loci appeared extra peaks. For instance, when the PCR products were stored for 4 days at room temperature, the real allelic peaks of loci DYS391 (10), DYS576 (16) and DYS458 (18) completely disappeared, which were replaced by their corresponding extra peaks and labeled as off-ladder (OL) and allele 18.1, respectively. At the same time, the EASPs were detected at loci DYS456, DYS460, DYS533, DYS635, and DYS449 etc. (Supplementary Figure S1). No artificial peaks were observed at any loci when the PCR products of the Y43CS were stored at − 20°C for 1–7 days.

When the amplification products of VFP were deposited continuously at three different temperature conditions for 10 days, no EASPs were seen at any loci. The peak heights of those loci with longer amplification products in the VFP decreased considerably when the products were stored at room temperature for 7 days, although no allele drop-out events were observed (Supplementary Figure S2).

### 3.2 Exploration of the cause of the EASP formation

#### 3.2.1 Variations of the amplification or electrophoresis conditions

To verify whether this EASP was formed by the secondary structure of amplified DNA fragments which was generated owing to inadequate denaturation before electrokinetic injection, 1.0 μL of PCR product that had been placed at room temperature for 5 days was added into 24.0 μL of loading mixture, followed by a heat denaturation at 95°C for 3 min. The findings suggested that increasing the volume of loading mixture and heating treatment could not eliminate the EASPs at the loci DYS391, DYS576 and DYS458 etc. (Supplementary Figure S3).

**FIGURE 3:**
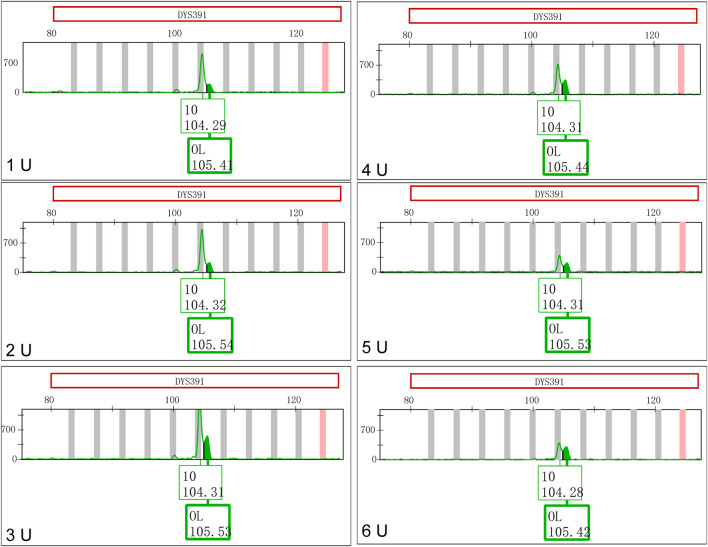
Increasing *Taq* DNA polymerases favor formation of excessive non-templated nucleotide addition products at locus DYS391.

The concentrations of *Taq* DNA polymerase in the Y43CS with a 10.0 μL reaction volume were varied from 1 U to 6 U. The PCR products were placed at room temperature for one day to observe the appearances and changes of EASPs. As shown in Figure 3, the peak height ratios of artificial peaks to main peaks at locus DYS391 increased gradually when the concentrations of DNA polymerases increased. The peak height ratios were 0.2119, 0.2272, 0.3871, 0.4746, 0.5414 and 0.7618 at the concentrations of 1 U, 2 U, 3 U, 4 U, 5 U and 6 U, respectively. The appearances and changes of EASPs at loci DYS576 and DYS458 were similar to those observed at locus DYS391, the difference was that the artifact appeared as a shoulder peak on the right side of the main peak. When the concentration of *Taq* DNA polymerase increased to 6 U, the extra peak appeared at locus DYS576 was labeled as an OL peak (Supplementary Figure S4). Except for loci DYS391, DYS576 and DYS458, no additional peaks appeared at other loci in the polymerase variation experiments, so only these three loci were displayed in Figure 3 and Supplementary Figure S4.

The effect of final extension on the generation of EASP in the Y43CS was implemented by varying the final extension time adopted in the PCR thermocycling program. All PCR products were maintained at − 20°C until electrophoresis on a 3500xL instrument for analysis and electrophoresed as per the standard electrophoresis condition of Y43CS. The effect could be seen in Supplementary Figures S5 and S6. The degrees of excessive nucleotide addition at loci DYS391, DYS460, DYS576, DYS458 and DYS570 etc. were gradually enhanced with the increasing of final extension time as expected.

#### 3.2.2 Termination of the PCR amplification reaction

**FIGURE 4:**
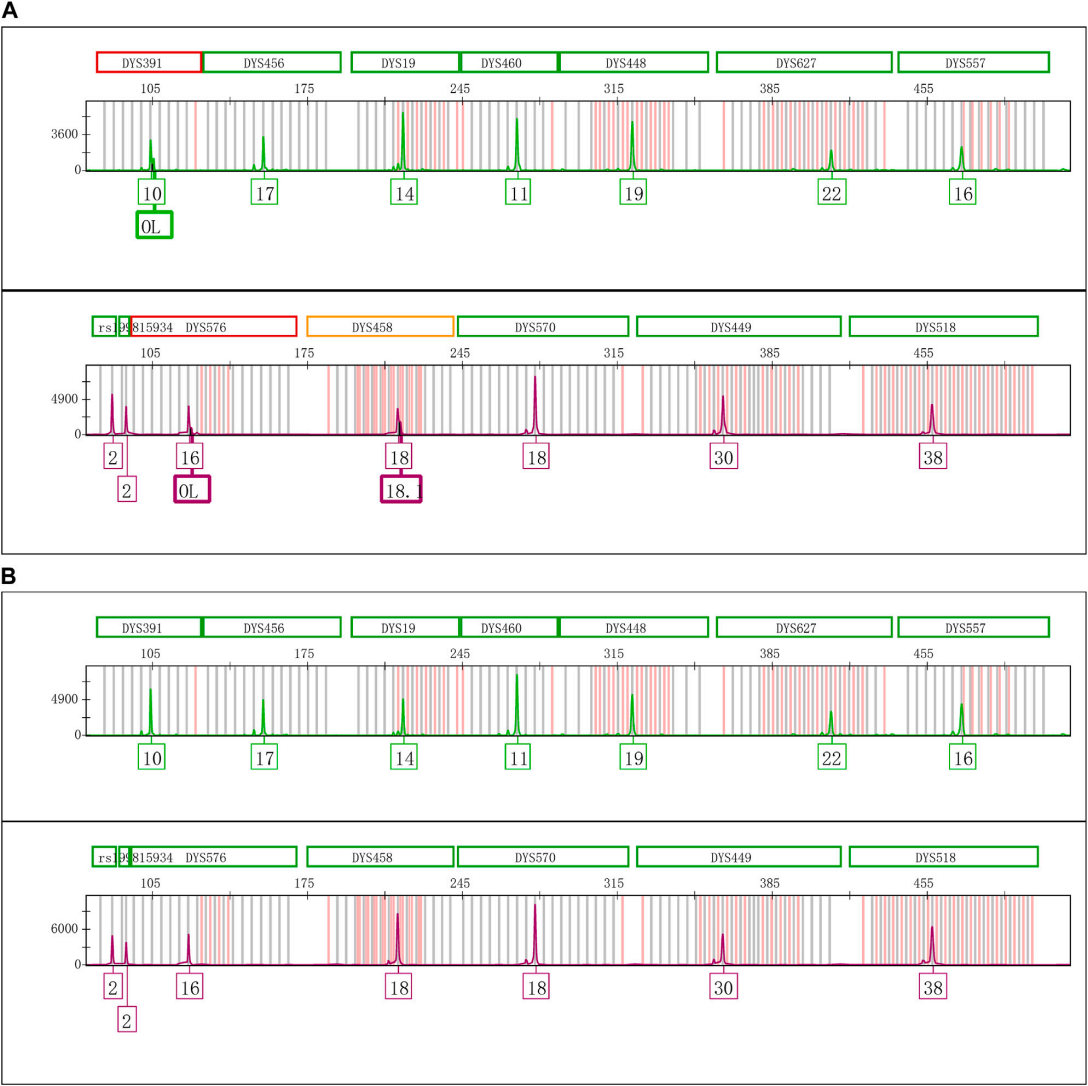
Electrophoretogram results of PCR products adding various concentrations of EDTA. **(A)** The PCR products with 1.0 mmol/L EDTA placed 6 days at room temperature. **(B)** The PCR products with 2.0 mmol/L EDTA placed 6 days at room temperature.

Termination of the PCR reaction was achieved by adding various concentrations of EDTA to the amplification products. The results of addition EDTA were shown in Figure 4, the EASPs were observed at loci DYS391, DYS576 and DYS458, when the PCR products with the addition of 1.0 mmol/L EDTA were stored at room temperature for 6 days. No extra peaks were detected at any loci regardless of the temperature at which the PCR products were stored when the final concentrations of EDTA exceeded 2.0 mmol/L. Additional experiment was performed to terminate the PCR amplification reaction by adding high volumes of formamide. Once the PCR amplification products mixed with excess formamide, no additional peaks were observed even after they were placed at room temperature for 10 days (Supplementary Figure S7).

### 3.3 Influences of modified primers on the overall performance of Y43CS

A total of 14 reverse primers were specifically modified among loci DYS391, DYS576, DYS458, DYS19, and DYS481 in the Y43CS (Table 1). The high specificity and amplification efficiency of each revised primer were initially confirmed by amplifying male (Control DNA 9948) and female (Control DNA 9947A, Promega Corporation) templates with singleplex PCR reactions. For each modification primer pair, i.e., the newly revised reverse primer mixed together with its corresponding original forward primer in Y43CS, a single amplicon peak could be detected at the expected size position. Each modification primer pair was incorporated separately into the Y43CS multiplex system when it was successfully confirmed. In the presence of revised reverse primers, instead of original reverse primers in Y43CS, complete profiles were obtained with male DNA were used as templates (Supplementary Figure S8). And no non-specific amplification products were found. The result demonstrated that the overall performance of the Y43CS was not significantly affected by modification of primers.

### 3.4 Effects of primer terminal nucleotide on EASP

By examining the primer sequences of all loci in the Y43CS, we found that the first two nucleotides at the 5′ end of the reverse primers of loci DYS391, DYS576 and DYS458 were AT. Two sets of control experiments were carried out to investigate the effect of AT nucleotides on the EASP formation of amplified products for the Y43CS. In one set of experiments, the AT nucleotides were removed from the reverse primers of loci DYS391, DYS576 and DYS458. The AT nucleotides were intentionally added to the 5′ end of the reverse primers of loci DYS19 and DYS481 in another set of experiments. PCR amplification products from all experiments were stored at 4°C and room temperature for 1 day.

**FIGURE 5:**
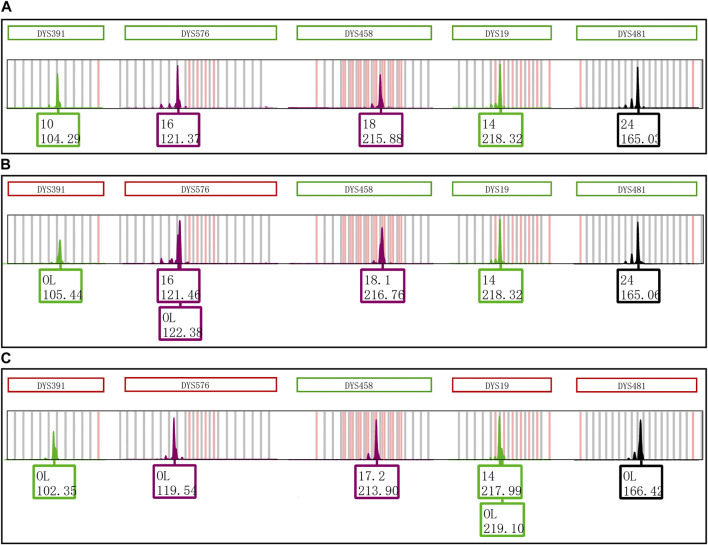
Primer modifications strongly affect the EASP formation. **(A)** The PCR products of original primers stored at 4°C for 1 day. Only one typical allelic peak with an expected peak size was observed at loci DYS391, DYS576, DYS458, DYS19 and DYS481, respectively. **(B)** The PCR products of original primers stored at room temperature for 1 day. The extra peaks appeared at loci DYS391, DYS576 and DYS458, but not observed at DYS19 and DYS481 loci. **(C)** The PCR products of revised primers stored at room temperature for 1 day. The reverse primers of loci DYS391, DYS576, DYS458, DYS19 and DYS481 were revised as described in Table 1. The phenomenon observed in Figure 5C is opposite to that of observed in Figure 5B. The extra peaks appeared at loci DYS19 and DYS481, but not observed at loci DYS391, DYS576 and DYS458.

The effect of nucleotides AT on the formation of EASP is shown in Figure 5. The EASPs were not observed at loci DYS391, DYS576 and DYS458 in PCR products which were amplified with the revised primers. On the contrary, the EASPs appeared at DYS19 and DYS481 loci when the AT nucleotides were added to the reverse primers of these loci. Especially for DYS481 locus, only EASP was observed after PCR products were placed at room temperature for 1 day. These findings demonstrated that the formation of EASP was strongly influenced by AT nucleotides at the 5′ end of the reverse primer.

**FIGURE 6:**
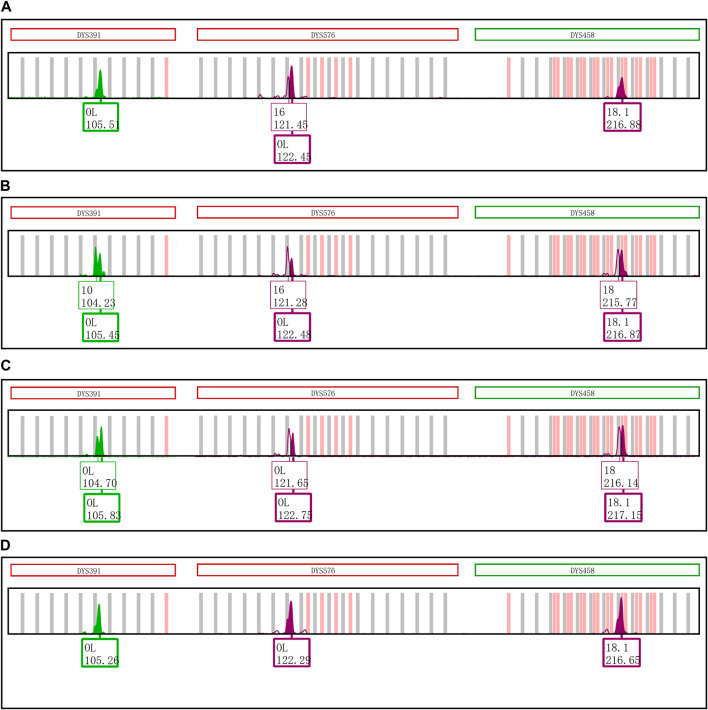
Changing the initial nucleotide at the 5′ end of reverse primers changes the proportion of excessive addition products. **(A)** PCR products of loci DYS391, DYS576 and DYS458 amplified by the original revers primers with an initial nucleotide A. **(B)** PCR products of loci DYS391, DYS576 and DYS458 amplified by the revised revers primers with an initial nucleotide T. **(C)** PCR products of loci DYS391, DYS576 and DYS458 that was amplified by the revised revers primers with an initial nucleotide C. **(D)** PCR products of loci DYS391, DYS576 and DYS458 that was amplified by the revised revers primers with an initial nucleotide G.

Furthermore, the first nucleotide A at the 5′ end of reverse primers for loci DYS391, DYS576 and DYS458 was modified as described in Table 1. And then PCR products were stored at 4°C and room temperature for 1 day to observe EASP. Replacing the A in position 1 of these reverse primers with T, C or G all caused some variations in the formation of EASPs. In general, 38%–100% PCR products were labeled as EASPs when the first nucleotide at 5′ end of reverse primers was changed from T to A, C or G (Table 2). The nucleotide G had the strongest potency to generate EASP followed by nucleotides A, C and T in our experiments. Almost all amplified products were converted into EASPs when the nucleotide G or A was positioned in the initial position of the 5′ end of the reverse primers (Figure 6).

**TABLE 2:** Primer modifications which influence formation of non-templated nucleotide addition products.

Loci	5′ end nucleotides^a^	Number of detected peaks	Size of peaks		Peak height ratio^b^	% ENA^c^	Ability to promote ENA^d^
DYS391	**A**T	one		105.51		100.00	G = A > C > T
	**T**T	two	104.23	105.45	0.7858	44.00	
	**C**T	two	104.70	105.83	1.4531	59.24	
	**G**T	one		105.26		100.00	
DYS576	**A**T	two	121.46	122.38	1.4759	59.64	G > A > C > T
	**T**T	two	121.20	122.40	0.4847	37.61	
	**C**T	two	121.66	122.86	0.8455	45.50	
	**G**T	one		122.28		100.00	
DYS458	**A**T	one		216.76		100.00	G = A > C > T
	**T**T	two	215.77	216.76	0.8150	49.13	
	**C**T	two	216.10	217.21	1.0739	51.36	
	**G**T	one		216.65		100.00	

Notes.

^a^ The nucleotides at the 5′ end of reverse primers and the changed nucleotides are in bold font.

^b^ The peak height ratio of peak with a larger size to peak with a smaller size.

^c^ Percentage of excessive nucleotide addition (ENA) is calculated by dividing the height of the ENA peak by the sum of the heights of the ENA and true peaks.

^d^ The ability of the base at the 5′ end of reverse primer to promote excessive nucleotide addition (ENA).

## 4 Discussion

Here, we observe one kind of artifact that preferentially appears at locus DYS391 in Y43CS amplification products, which preserve at 4°C for a few days. A significant difference between this artifact and previously defined split peak is that the size of this extra peak is about one base larger than the true allele and locates on the right of the real allelic peak. Our findings indicate that the excessive non-templated nucleotide addition artificial peak presumably formed by the terminal transferase activity of *Taq* DNA polymerase after PCR amplification.

There are at least two possible mechanisms for the formation of this artificial peak: (i) the secondary structure of the PCR amplified products (Bovo et al., 1999), (ii) the results of non-template extension by *Taq* DNA polymerase (Smith et al., 1995). Experiments were performed to verify the first hypothesis that the accidentally observed artifact is the secondary structure of PCR fragment. Firstly, the amplicon sequences of loci DYS19 (Accession Number, AN: AC017019), DYS576 (AN: AC010104) and DYS458 (AN: AC010902) in the Y43CS were analyzed by BioXM v2.7 and RNAstructure v6.1 software packages. Analysis results showed that the molecular structures of these loci do not exhibit the characteristics to form secondary structure preferentially (data not shown). Moreover, the following experimental results also show that this artifact cannot be eliminated by increasing loading mixture volume and conducting heat denaturation before injection. The results indicate that the extra peak may not be caused by double-stranded amplicons of these loci which formed under suboptimal electrophoresis conditions (McLaren et al., 2008; Zhou et al., 2020).

Another strategy is designed to confirm the second possible mechanism by termination of the PCR amplification reaction. In the PCR termination experiment, large volumes of formamide (Hutton, 1977) or high concentrations of EDTA (Lorenz, 2012) are added to the amplified products immediately, as soon as the PCR amplification finished. When PCR products without EDTA are placed at room temperature for 6 days, extra peaks are observed at 13 loci. Under the same storage conditions, extra peaks are observed at only three loci when the PCR products contained EDTA with a final concentration of 1.0 mmol/L. The peak height ratios of extra peaks to main peaks are 0.3975, 0.2609, and 0.5313 at loci DYS391, DYS576, and DYS458, respectively. However, only extra peaks are observed at these three loci in PCR products without EDTA. When the final concentration of EDTA in PCR products exceeds 2.0 mmol/L or 30.0 μL of formamide is added to 10.0 μL of PCR products, no additional peaks are observed at all loci.

These results demonstrate that the amplification reaction of Y43CS can be effectively inhibited or terminated by EDTA and formamide. As a chelating agent for divalent metal ions, EDTA is expected to deplete Mg^2+^ in a PCR mixture, and thus inhibit the activity of DNA polymerase (Schrader et al., 2012). It may also inhibit PCR amplification by changing the stability of double-stranded DNA (Thompson et al., 2014). Formamide is an important denaturing agent that can effectively denature proteins and nucleic acids (Shedlovskiy et al., 2017), as well as preserve RNA because of its ability to suppress RNase activity (Chomczynski, 1992). Therefore, it is likely that it terminates the PCR reaction by inactivating the *Taq* DNA polymerase. From the experimental results (Figure 4; Supplementary Figure S7), we can see that the extra peaks disappeared with the termination of PCR reaction. These results and the non-templated nucleotide addition feature of *Taq* DNA polymerase (Clark, 1988; Smith et al., 1995) support the second possible mechanism that the observed extra peaks in the present study are EASPs.

It is not surprising that the non-templated nucleotide addition reactions occur during nucleic acid replication *in vivo* (Hoang et al., 2017) or PCR amplification *in vitro* (Clark, 1988; Hu, 1993; Kolganova et al., 2018), but it is interesting that this event occurs after the PCR amplification. In our experiments, the addition of extra nucleotides occurs at the stage of PCR products storage. And the degree of nucleotide residues addition is affected by the storage time and temperature. For loci DYS391, DYS576 and DYS458 in the Y43CS, EASPs are observed when the PCR products are stored at 4°C for 6 days or room temperature for 1 day. A similar case is observed by V.L. Magnuson et al., 5%–25% allele changed to “+ A allele” in some PCR products stored at 4°C after 2 days (Magnuson et al., 1996).

One more interesting finding is that EASP is a + 2 addition product formed by *Taq* DNA polymerase adding two extra nucleotides to termini of nascent blunt end DNA fragment. This means that EASP is two nucleotides longer than the product predicted on the reference sequence. However, the “+ A allele” is a + 1 addition product which is one base longer than the expected amplicon (Clark et al., 1987; Magnuson et al., 1996; Butler, 2015). This is the major difference between EASP described in this study and previously reported “+ A allele” or split peak. In theory, the Y-STR alleles in Y43CS are all in the + 1 addition form by a final extension step at 60°C for 10 min (Brownstein et al., 1996; Magnuson et al., 1996). As shown in Figures 1 and 2, EASPs are sized approximately one base greater than the Y-STR alleles by GeneMapper^™^ ID-X. Based on these findings, we believe that the EASP is an artifact formed by the addition of two extra nucleotides onto the expected amplification product. Although DNA Polymerase I mutant can produce + 2 addition product too, the + 2 product is much less frequently observed than that of + 1 addition product (Clark et al., 1987). For some PCR products in this study, 100% of the PCR products can be converted to + 2 products at loci DYS391, DYS576 and DYS458 (Table 2; Supplementary Figure S1). This may be due to the superior terminal extendase activity of the *Taq* DNA polymerase (Hu, 1993; Terpe, 2013).

The AT nucleotides tail is pointedly added to the 5′ end of the reverse primers for loci DYS391, DYS576 and DYS458 in the Y43CS for the purpose to promote non-templated nucleotide addition of the amplification products. In the experiments, however, we note that they exhibit a relatively strong preference for the formation of EASP. As long as the two terminal nucleotides (AT) are removed from the reverse primers of these loci, their preference for forming EASPs is weakened significantly. The EASPs formation of Y-STR loci in the Y43CS is strongly affected by their primer sequences. Our findings are roughly similar to a previous report that claimed the degree to which marker add extra nucleotide is primer-specific (Smith et al., 1995). The efficiency of addition extra nucleotide by DNA polymerase is DNA terminal-dependent (Hu, 1993; Brownstein et al., 1996), which may also be appropriate for the EASP. Overall, the efficiency of the terminal nucleotide modification on stimulating EASP formation is different, with efficiencies in the order G ≥ A > C > T. These results are consistent with previous reports that *Taq* DNA polymerase can effectively add a non-templated extra nucleotide adjacent to a terminal substrate nucleotide C, but not to a base A (Hu, 1993; Brownstein et al., 1996; Magnuson et al., 1996).

The degree of excessive non-templated nucleotide addition follows previously identified rules that the degree of “plus A” modification is affected by final extension time, concentration of DNA polymerase and other factors (Smith et al., 1995). Both increasing *Taq* DNA polymerases and final extension times tend to increase the amounts of EASPs. The degree of excessive nucleotide addition has been increasing with the increase of the final extension time. This finding seems to be not entirely consistent with previous report that the final extension time of 90 min maximizes the non-templated nucleotide addition capacity of *Taq* DNA polymerase (Smith et al., 1995). It should be noted that no EASP is observed at any loci in the control kit VFP even though its PCR products are placed 10 days at room temperature. Although the underlying cause of the phenomenon is unknown, it is not hard to speculate that the components in PCR master mix might be contribute to this result, especially the hot start DNA polymerase used in VFP. Since the *Taq* DNA polymerase used in VFP and Y43CS are different, these experimental results indirectly indicate that different forms of *Taq* polymerase may have different effects on the long-term preservation of PCR products.

Multiplex short tandem repeat (STR) kits are commonly used in the field of forensic DNA analysis. Forensic geneticists and forensic DNA analysts are extremely strict about the accuracy and precision of the genotyping results produced by STR kits in their daily work. Even if the size of STR-PCR products differs by only one base, it is possible to have a serious effect on genotyping, resulting in incorrect typing results (Chen et al., 2013) which may even lead to the wrongful arrest or release of criminal suspects. In practical work, due to the need of case investigation, the STR-PCR products are usually detected at different time points several times to ensure the accuracy and reliability of the typing results. In the meantime, the STR-PCR products are routinely stored at 4°C, or room temperature, away from light.

Previously, we did not pay much attention to the phenomenon that STR-PCR products would form + 2 addition products during storage and lead to incorrect typing results. For example, the allele 9.3 at autosomal STR locus TH01 differs from the allele 10 by a single base deletion of adenine in the seventh repeat (Puers et al., 1993). If the genotyping result [9.3, 9.3] at locus TH01 for a biological sample/criminal suspect is changed to [10, 10] because of the + 2 addition products formed during preservation, the investigation of the case is very likely to go astray. One of the main objectives of this study is to inform forensic DNA analysts that STR-PCR products may produce false typing results due to the formation of + 2 addition products even under non-PCR amplification conditions. Therefore, measures should be taken to terminate PCR reaction immediately after the completion of PCR amplification, to effectively reduce the probability of genotyping errors and ensure the correctness of results and judicial fairness.

## 5 Conclusion

In this study, we described the characteristics of an extra peak unexpectedly observed in a Y-STR profile and explored the possible causes of it. This artifact is an excessive non-templated nucleotide addition product that is produced by *Taq* DNA polymerase. Although the PCR amplification reaction in the thermal cycler has been completed, the non-templated nucleotide addition still proceeds at 4°C or room temperature, and then forms EASP during the PCR products preservation. A significant difference between the EASP and the previously reported split peak is that the EASP is a + 2 addition product. Another difference is that the EASP is formed after PCR amplification. Finally, we suggest that the PCR products should be frozen or denatured after the PCR amplification reaction as quickly as possible, to keep the product intact and maximize correct genotype calls.

## Data Availability

The original contributions presented in the study are included in the article/Supplementary Material, further inquiries can be directed to the corresponding authors.

## Supplementary material

SUPPLEMENTARY FIGURE S1 Electrophoretogram results of Y43CS. A genotyping profile generated from 1.0 ng of Control DNA 9948 amplified with the Y43CS kit for 30 cycles and electrophoresed on an Applied Biosystems 3500xL Genetic Analyzer. The PCR products were placed at room temperature for 4 days. Additional peaks appearing at Y-STR loci were selected and marked as solid.

SUPPLEMENTARY FIGURE S2 Electrophoretogram results of VFP. A genotyping profile generated from 1.0 ng of Control DNA 9948 amplified with the VeriFiler^™^ Plus kit for 30 cycles and electrophoresed on an Applied Biosystems 3500xL Genetic Analyzer. The PCR products were placed at room temperature for 7 days. Y-STR loci with significantly reduced peak heights were selected and marked as solid.

SUPPLEMENTARY FIGURE S3 Electrophoretogram results of PCR products stored at room temperature for 5 days. Additional peaks appearing at Y-STR loci were selected and marked as solid.

SUPPLEMENTARY FIGURE S4 Increasing Taq DNA polymerases which favor formation of excessive non-templated nucleotide addition products. The results are shown in order of 1 U to 6 U from top electrophoretogram to bottom electrophoretogram for loci DYS576 and DYS458.

SUPPLEMENTARY FIGURE S5 Electrophoretogram of green dye channel in the final extension time variation experiment. Y-STR loci with obvious extra peaks were selected and marked as solid. The results are shown in order of 0 min to 120 min from top electrophoretogram to bottom electrophoretogram.

SUPPLEMENTARY FIGURE S6 Electrophoretogram of purple dye channel in the final extension time variation experiment. Y-STR loci with obvious extra peaks were selected and marked as solid. The results are shown in order of 0 min to 120 min from top electrophoretogram to bottom electrophoretogram.

SUPPLEMENTARY FIGURE S7 Electrophoretogram results of PCR products added 30.0 μL of formamide.

SUPPLEMENTARY FIGURE S8 Control DNA 9948 profile with revised reverse primer of locus DYS391. The 5′ end initial nucleotide A of the original reverse primer was replaced by nucleotide G. The modified reverse primer was used to replace the original reverse primer in the Y43CS to establish a new primer set. A representative profile generated from 1.0 ng of Control DNA 9948 amplified with the new primer set for 30 cycles and electrophoresed on an Applied Biosystems 3500xL Genetic Analyzer.
