# Supplementary figures and images for: Excessive addition split peak formed by the non-templated nucleotide addition property of Taq DNA polymerase after PCR amplification
Source: Front Bioeng Biotechnol. 2023 Apr 27;11:1180542. doi: 10.3389/fbioe.2023.1180542 (PMC10174434; doi:10.3389/fbioe.2023.1180542)

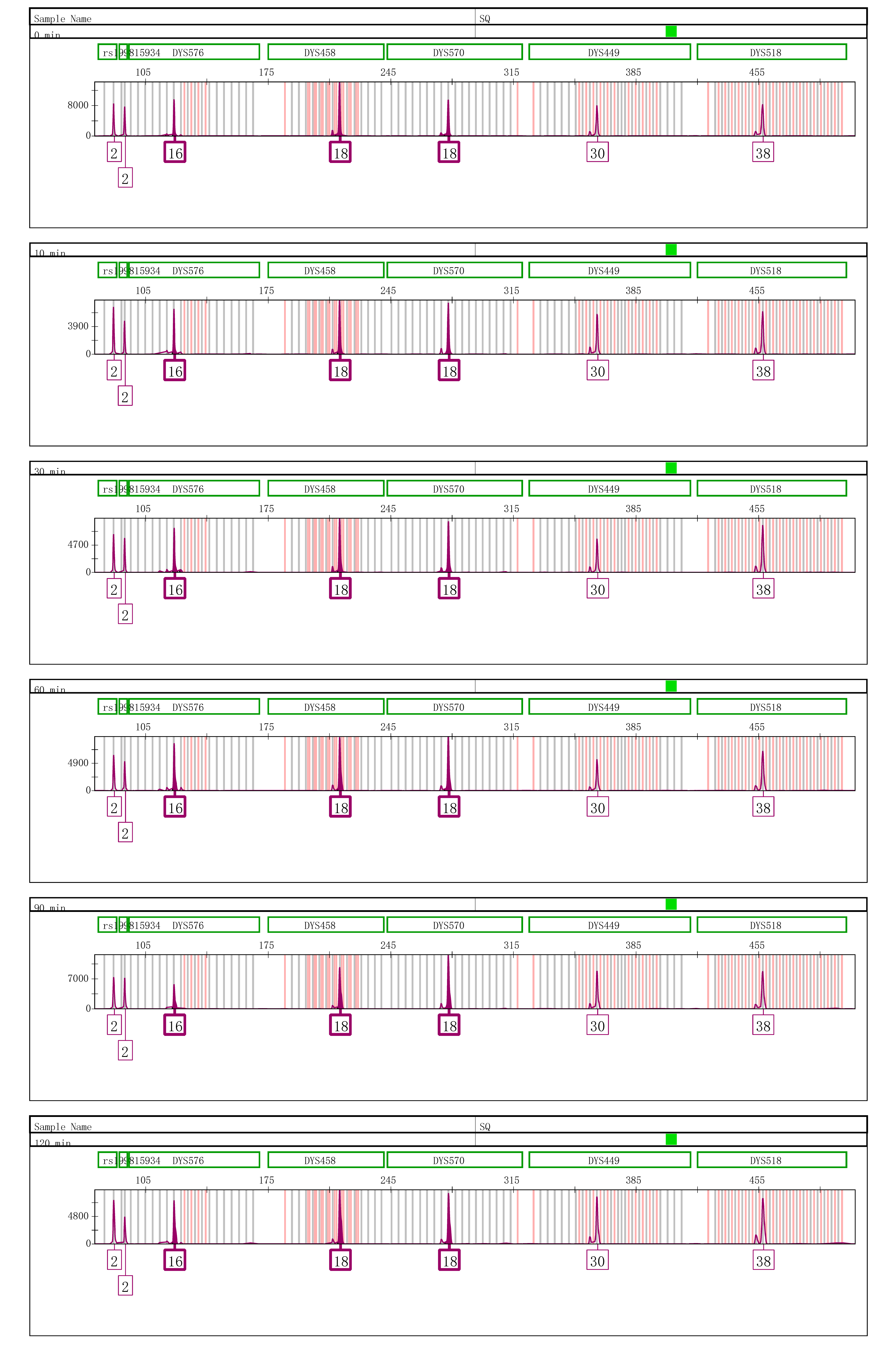

Supplement: Supplementary file 2 [file Image6.TIF]

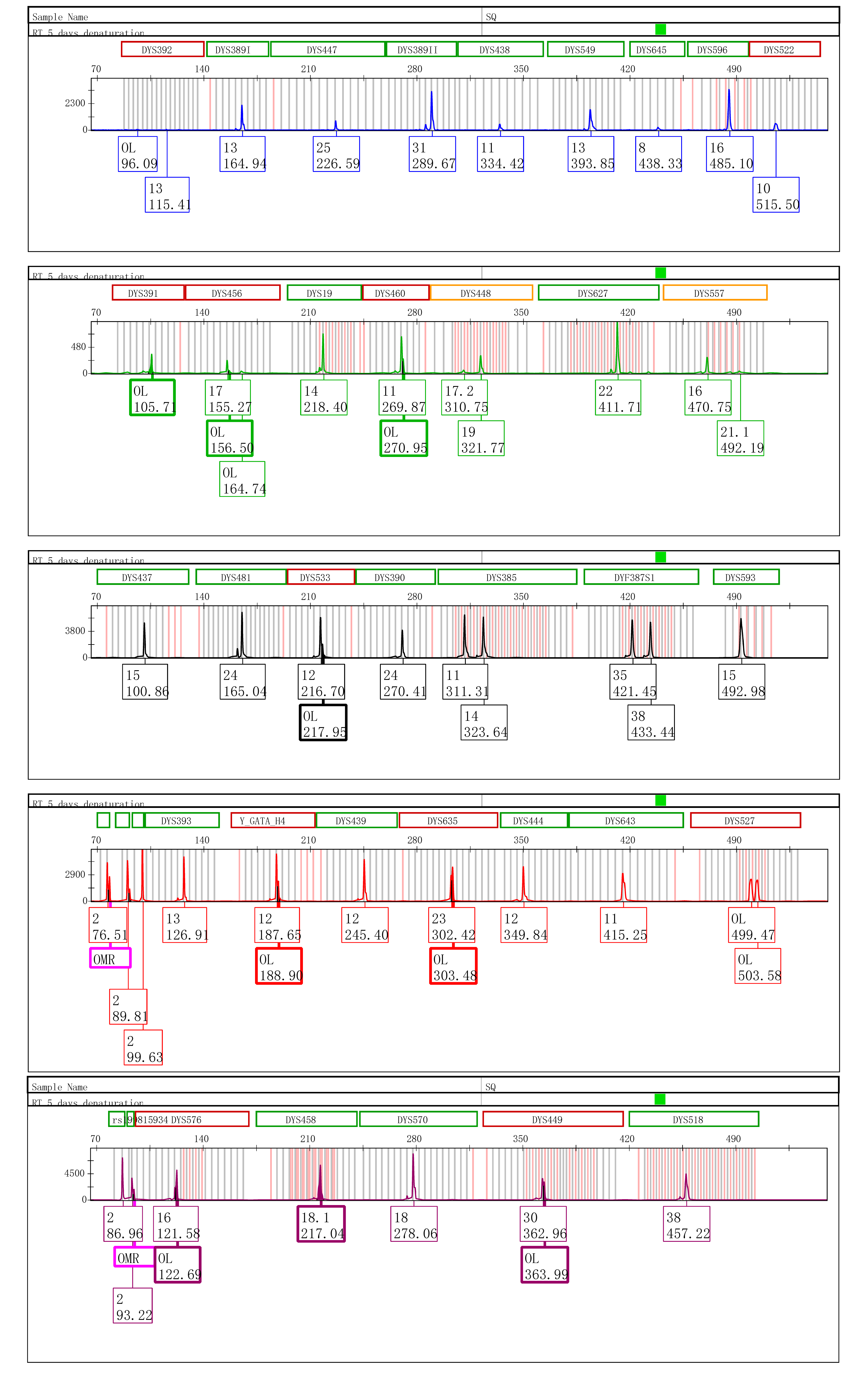

Supplement: Supplementary file 3 [file Image3.TIF]

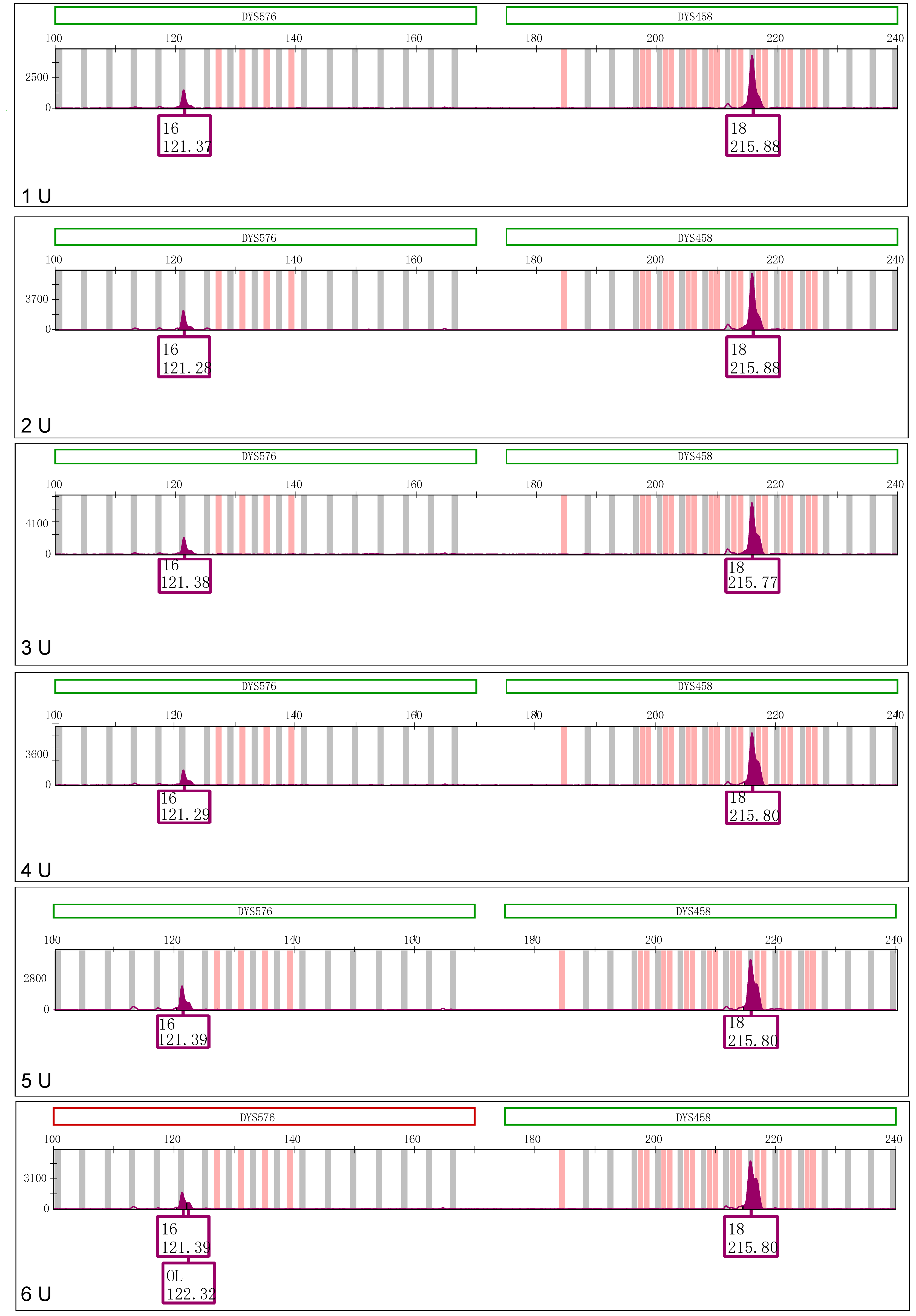

Supplement: Supplementary file 4 [file Image4.TIF]

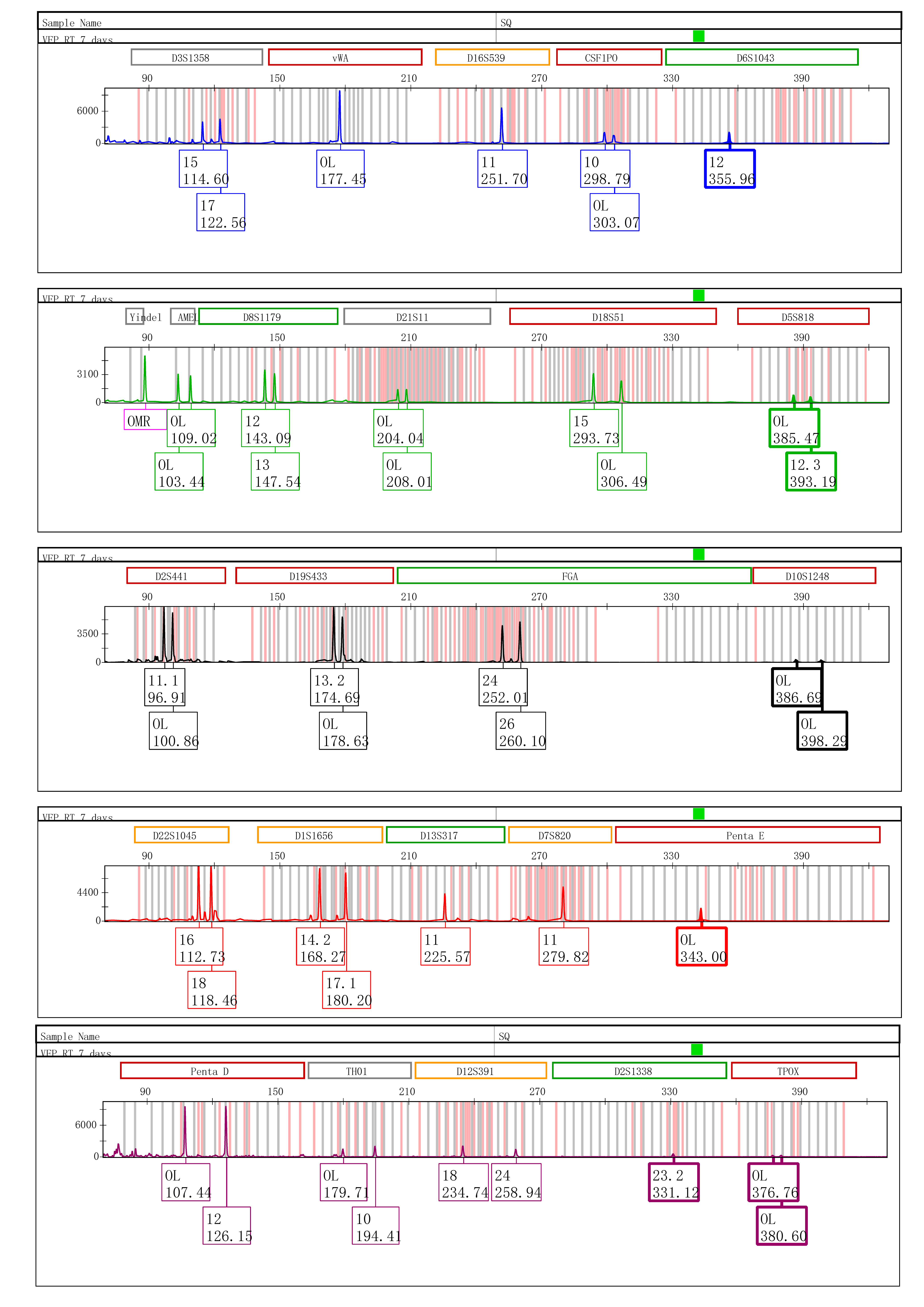

Supplement: Supplementary file 5 [file Image2.TIF]

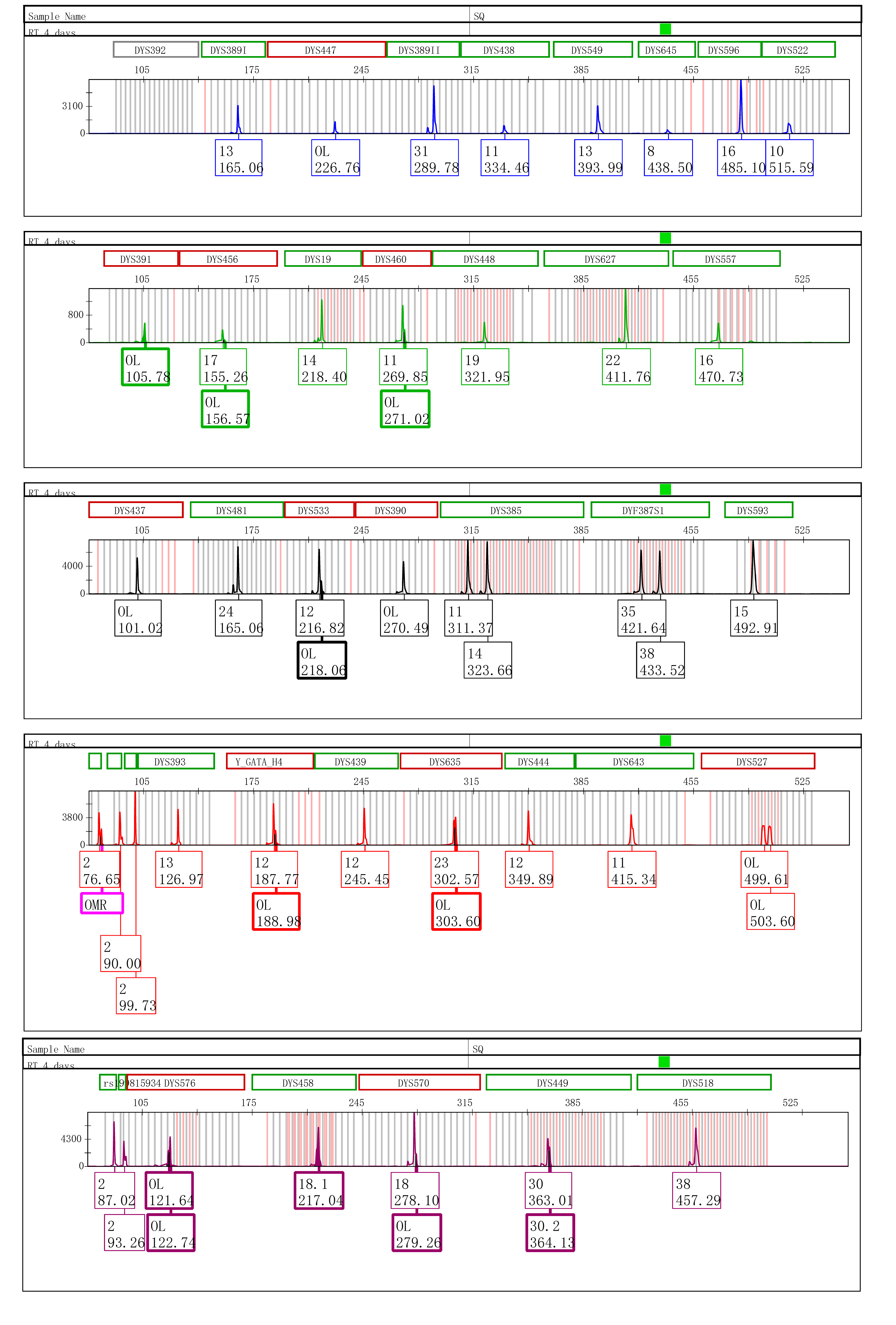

Supplement: Supplementary file 6 [file Image1.TIF]

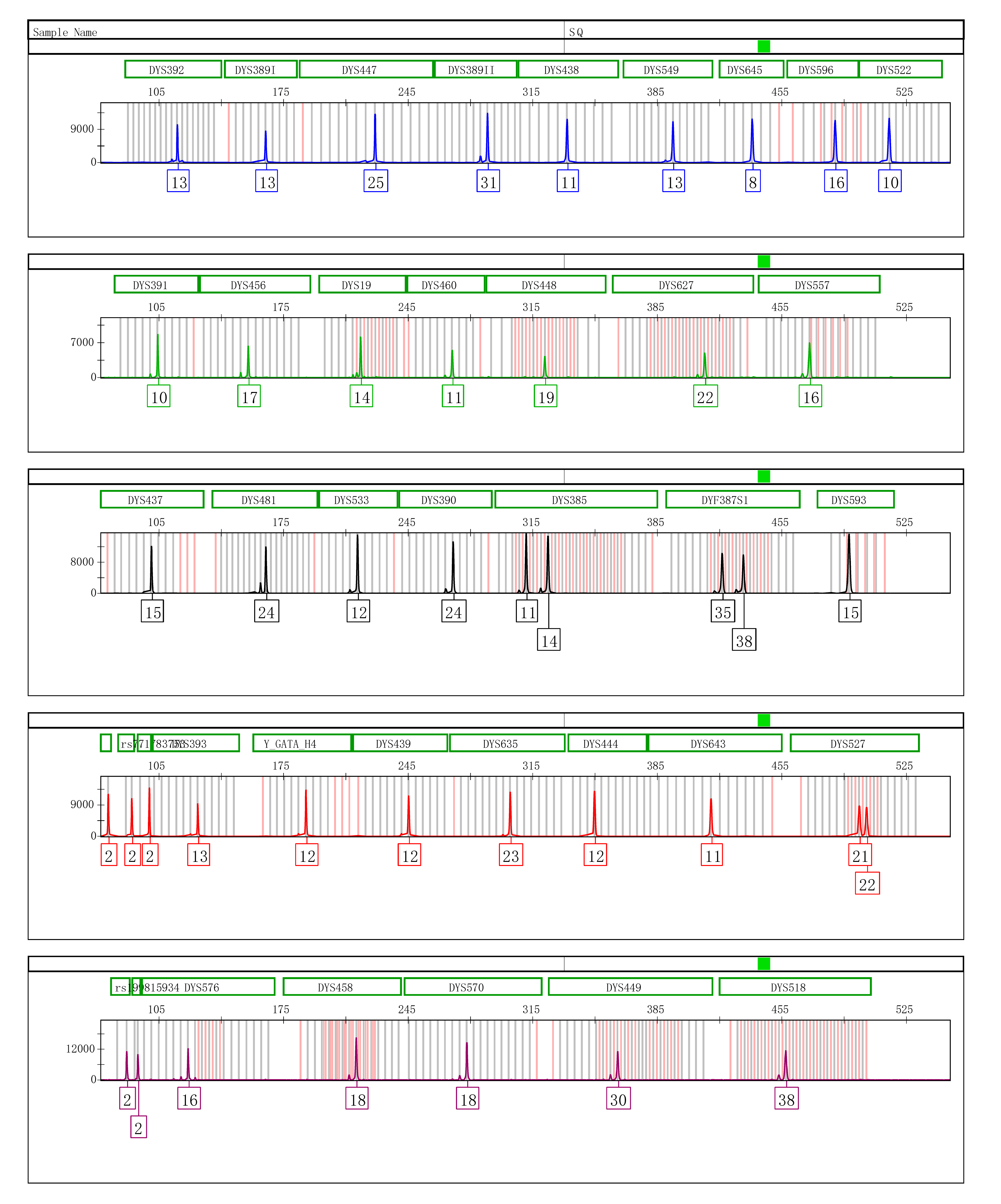

Supplement: Supplementary file 7 [file Image7.TIF]

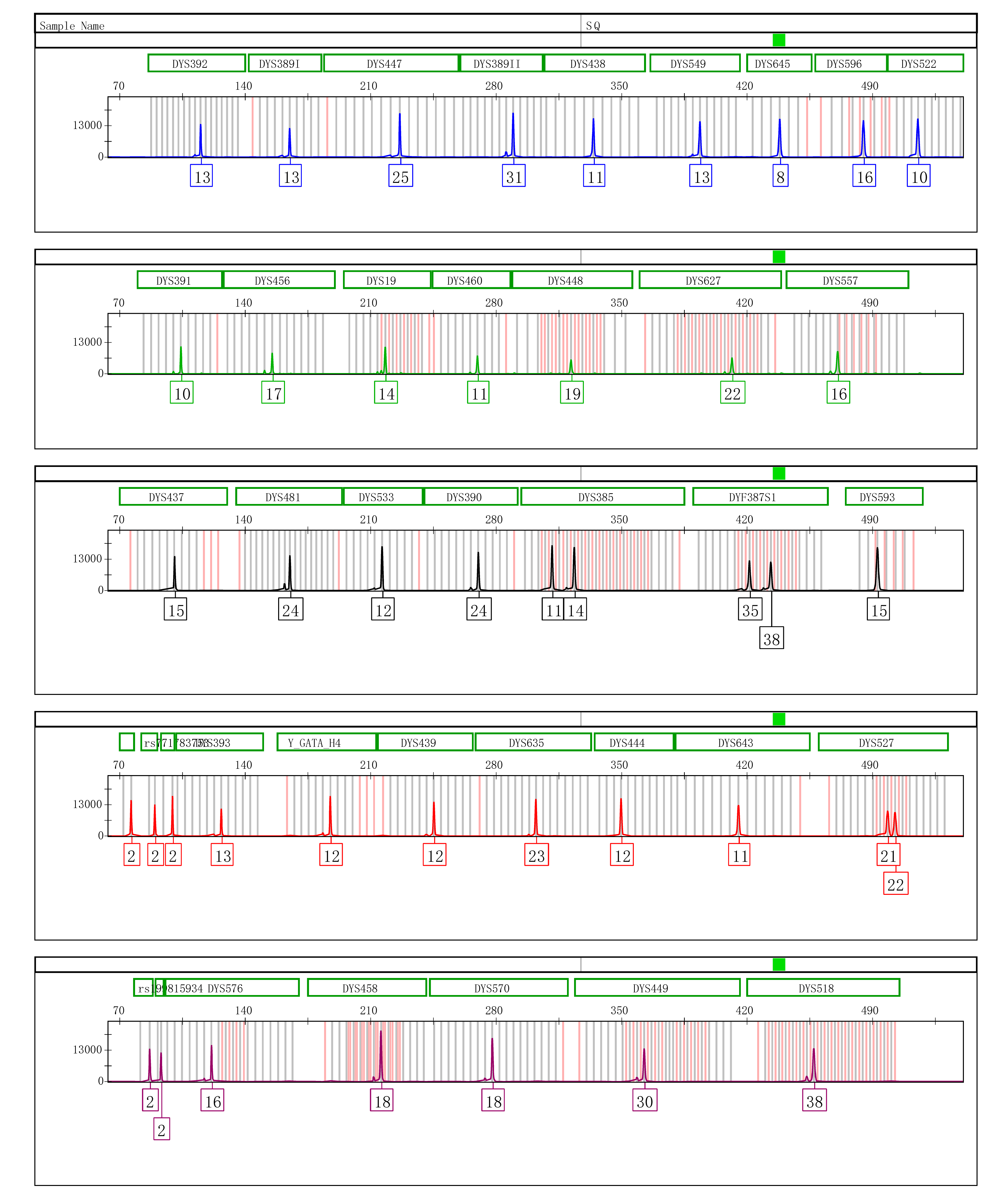

Supplement: Supplementary file 8 [file Image8.TIF]

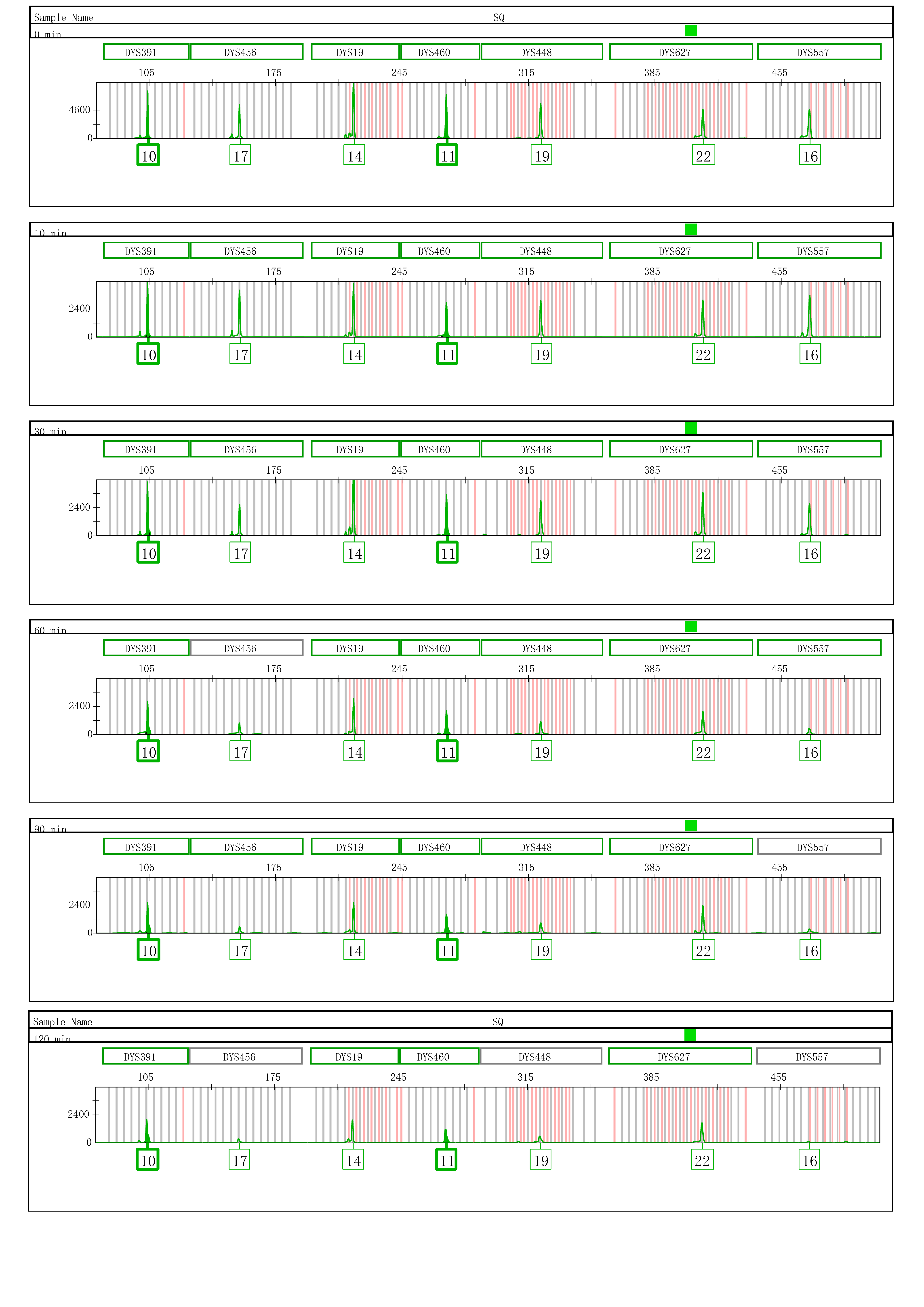

Supplement: Supplementary file 9 [file Image5.TIF]
